# Outbreak of gastrointestinal anthrax following eating beef of suspicious origin: Isingiro District, Uganda, 2017

**DOI:** 10.1371/journal.pntd.0008026

**Published:** 2020-02-27

**Authors:** Miriam Nakanwagi, Alex Riolexus Ario, Leocadia Kwagonza, Freda Loy Aceng, James Mwesigye, Lilian Bulage, Joshua Buule, Juliet Nsimire Sendagala, Robert Downing, Bao-Ping Zhu

**Affiliations:** 1 Uganda Public Health Fellowship Program-Field Epidemiology Track, Kampala, Uganda; 2 Department of Microbiology, Mbarara Regional Referral Hospital, Mbarara, Uganda; 3 UVRI-Abbott Research Laboratory, Uganda Virus Research Institute, Entebbe, Uganda; 4 MRC/LSHTM/UVRI, Uganda Virus Research Institute, Entebbe, Uganda; 5 Global Health Security Agenda, Uganda Virus Research Institute, Entebbe, Uganda; 6 US Centers for Disease Control and Prevention, Kampala, Uganda; 7 Division of Global Health Protection, Center for Global Health, US Centers for Disease Control and Prevention, Atlanta, United States of America; National Institutes of Health, UNITED STATES

## Abstract

**Introduction:**

Gastrointestinal anthrax is a rare but serious disease. In August 2017, Isingiro District, Uganda reported a cluster of >40 persons with acute-onset gastroenteritis. Symptoms included bloody diarrhoea. We investigated to identify the etiology and exposures, and to inform control measures.

**Methods:**

We defined a suspected case as acute-onset of diarrhoea or vomiting during 15–31 August 2017 in a resident (aged≥2 years) of Kabingo sub-county, Isingiro District; a confirmed case was a suspected case with a clinical sample positive for *Bacillus anthracis* by culture or PCR. We conducted descriptive epidemiology to generate hypotheses. In a case-control study, we compared exposures between case-patients and neighbourhood-matched controls. We used conditional logistic regression to compute matched odds ratios (MOR) for associations of illness with exposures.

**Results:**

We identified 61 cases (58 suspected and 3 confirmed; no deaths). In the case-control study, 82% of 50 case-patients and 12% of 100 controls ate beef purchased exclusively from butchery X during the week before illness onset (MOR = 46, 95%CI = 4.7–446); 8.0% of case-patients and 3.0% of controls ate beef purchased from butchery X and elsewhere (MOR = 19, 95%CI = 1.0–328), compared with 6.0% of case-patients and 30% of controls who did not eat beef. *B*. *anthracis* was identified in two vomitus and one stool sample. Butchery X slaughtered a sick cow and sold the beef during case-patients’ incubation period.

**Conclusion:**

This gastrointestinal anthrax outbreak occurred due to eating beef from butchery X. We recommended health education, safe disposal of the carcasses of livestock or game animals, and anthrax vaccination for livestock.

## Author summary

Gastrointestinal anthrax is one of the forms of anthrax caused by *Bacillus anthracis*. It is a serious but rarely diagnosed form owing to its presentation being similar to gastroenteritis illness due to other organisms. In this investigation, we ascertained that this was a gastrointestinal anthrax outbreak that was caused by consumption of beef of suspicious origin. Trace back identified the source of this beef as being from one specific butcher. All the case-patients were treated and recovered. The outbreak was controlled at the source through treatment and we provided public education of the masses about the dangers of eating meat of animals that have died of unknown causes. We additionally recommended inspection of meat before sale at butcheries to ensure that it is suitable for human consumption as well as vaccination of the animals in the region. In the long term, anthrax control requires a One-Health approach that is multisectoral, involving ministries of agriculture, animal industries and ministries of health.

## Introduction

Anthrax is a zoonotic bacterial disease that occurs worldwide, with an estimated 2,000 to 20,000 human anthrax cases occurring each year [1]. The causative agent of anthrax, *Bacillus anthracis*, is a large, gram-positive, aerobic, spore-forming bacillus [2, 3]. The infectious form of *B*. *anthracis* is usually the spores, which can remain viable in the environment for an extended period of time [1, 4, 5] even under extreme environmental conditions [6]. Although the global incidence has reportedly declined, cases are still common in African and Asian regions [2]. Frequent interactions between humans and animals during agricultural activities in these regions are often associated with human infections [7].

Human anthrax infections are classified into cutaneous, gastrointestinal, and inhalational disease, depending on the clinical features and transmission routes [3, 7]. Human gastrointestinal anthrax occurs after ingesting meat and other products from infected animals [3, 6, 7, 8, 9]. The incubation period ranges from 1–6 days, with a median of 3 days [3, 10, 11, 12]. Patients commonly present with fever, malaise, abdominal pain and tenderness, diarrhoea, nausea, vomiting, and abdominal distention. In severe cases, there may be hemorrhage (commonly manifesting as bloody diarrhoea), intestinal obstruction or perforation, ascites, sepsis, or shock [2, 3, 6, 10,11]. Gastrointestinal anthrax can be confirmed by positive culture from vomitus, stool, blood, peritoneal or tissue samples, or by positive serology [3]. Even with treatment, mortality may reach 40% [5].

In Uganda multisectoral committee on the prioritization of zoonotic diseases ranked anthrax as the highest-priority zoonotic disease based on its frequency in the country, among other factors [13]. However, there has been minimal documentation of the anthrax outbreaks in humans in Uganda.

On 23 August, 2017, Mbarara Regional Referral Hospital (MRRH) reported to the Uganda Ministry of Health (UMoH) that more than 40 patients with acute gastroenteritis had been referred from Rwekubo Health Centre in Isingiro District in a single day. Patient symptoms included abdominal pain, vomiting, and acute diarrhoea. Some of the patients had bloody diarrhoea. Based on the patients’ symptoms and his professional experience, the attending physician suspected it might be gastrointestinal anthrax and immediately administered intravenous antibiotics to all patients. We investigated this outbreak to determine its etiology, assess its scope and risk factors, and recommend control measures.

## Methods

### Setting

Isingiro District (0.84° S, 30.80° E) is located in southwestern Uganda. The district had an estimated population of 490,000 in 2017 based on projections from the 2014 census [14]. The climate is tropical savannah with an average annual rainfall of 1200mm, and temperature range of 17–30°C. The district has two rainy seasons in each year: March to April, and September to November [15]. Isingiro District is one of the major producers of indigenous cattle and goats in Uganda, and 52% of the population is engaged in livestock farming. The ecological system is prone to long periods of drought. The vegetation includes thorny bushy trees, grassland savannah, scattered swamps, and bare stony hills [16]. Most residents are affected by poverty and poor health [16].

### Case definition and case finding

We defined a suspected case-patient as acute-onset of diarrhoea (≥3/day) or vomiting between August 15 and 31, 2017 in a resident of Kabingo sub-county, Isingiro District. A confirmed case-patient was a suspected case-patient with a stool, blood, or vomitus sample positive for *B*. *anthracis* by culture and PCR. To identify case-patients, we reviewed inpatient files at MRRH and Rwekubo Health Centre for patients admitted during August 15–31, 2017. We also searched for suspected case-patients in outpatient records. From the patient files, we abstracted data on age, sex, place of residence, date of admission, clinical presentation including symptoms and signs of gastrointestinal, cutaneous and inhalational anthrax, duration of symptoms, treatment, laboratory findings, status at discharge, and date of discharge. We also worked with members of the Village Health Team to actively search for case-patients in villages of Kabingo sub-county using the case definition. We elicited history of contact with dead animals during butchering, skinning and meat preparation and additionally looked for patients with symptoms of cutaneous and inhalational anthrax.

### Descriptive epidemiology

We described case-patients by person, place, time, and clinical presentation, and calculated attack rates (AR) by age and sex using the 2017 projected population data for the villages in Kabingo sub-county, based on the 2014 Uganda Population and Housing Census. We constructed case-cluster maps depicting the case-patients’ village centers (actual case-patient addresses were not available). Using the epidemic curve, we described the course of the outbreak. For the start date, we counted back the minimum incubation period (from the date of illness onset of the first case) or median incubation period (from the peak of the epidemic curve), selecting the earlier date. For end date, we counted back the maximum incubation period (from the date of illness onset of the last case-patient). Using the calculated start and end date, we estimated the period of likely exposure.

In early interviews of case-patients, several reported eating beef before becoming ill suggesting that eating beef could be an exposure of interest.

### Case-control study

We conducted a matched case-control study. We recruited 50 case-patients and two asymptomatic neighborhood-matched controls per case. We defined a control as a resident of Kabingo sub-county over 2 years of age without acute onset of diarrhoea or vomiting during August 15–31, 2017.

We used a standardized questionnaire to interview case-patients and controls and collect demographic data, including age, sex, place of residence, and occupation, as well as data on eating beef and other cattle products such as milk and ghee, the source of the beef, and the method of beef preparation. As Isingiro District is one of the major producers of indigenous cattle and goats in Uganda with half of the population raising livestock, we also inquired about animal vaccination practices including anthrax vaccination.

To account for the matched case-control study design, we used conditional logistic regression to assess the associations between potential exposures and illness, calculating matched odds ratio (MOR) with 95% confidence interval (95% CI) for each exposure. Matched case-control sets were the units of analysis.

### Trace-back investigation

We investigated the single business that on trace-back sold the epidemiologically implicated food. We visited this business, and spoke with neighbours and shop owners in the area about the implicated food item to inquire about food sourcing and selling practices. This identified a single farm as the source of the food item; we visited this farm and interviewed the proprietors and other relevant authorities in the district about relevant events surrounding this food item before its sale.

### Laboratory investigations

We took clinical specimens (including eight blood, three vomitus, and three stool samples) from eight case-patients admitted at MRRH, before administration of antibiotics. Additionally, we collected blood and stool from a cow that had bloody diarrhoea on the implicated farm. The clinical laboratory at MRRH conducted the initial microscopic examination of the blood, vomitus, and stool specimens of some case-patients by gram staining. An initial culture was also done in MRRH on Robertson cooked meat medium and sub-cultured on blood and chocolate agar at 37°C for 24 hours. Initial microscopic examination of the gram stain of one vomitus sample showed a gram-positive, rod shaped bacterium suggesting possible anthrax. Growth of greyish white colonies which on gram stain showed gram-positive rods with spaces in between them further increased the suspicion of anthrax. Further processing of the samples at MRRH was then immediately stopped because the laboratory did not meet certification for further procedures and tests with anthrax. We inoculated the blood, vomitus and stool samples into brain heart infusion broth enrichment media and transported them to the Uganda Virus Research Institute (UVRI), a Biosafety level 3 laboratory for further processing. At UVRI, the vomitus and blood samples were sub-cultured on sheep blood agar incubated at 37°C in Carbon Dioxide for 24hours. Stool specimens were cultured in selenite F selective broth and after overnight incubation at 37°C in air, they were sub cultured on MacConkey agar in air and blood agar in Carbon Dioxide both at 37°Cc for 24 hours. Suspected colonies were gram stained and examined microscopically. Human samples yielding gram-positive rod-shaped bacilli by culture were further investigated using polymerase chain reaction (PCR) at the UVRI and the National Animal Diseases Diagnostics and Epidemiology Centre for PCR testing. Fresh culture of B. *anthracis* from luria broth sub-cultured from blood agar was used for DNA extraction for genomic and plasmid DNA extraction using commercially available DNA IQ^™^ system (Promega corporation, USA) following manufacturer’s instructions. PCR for amplification of pXO1 plasmid targeting protective antigen, pXO2 plasmid targeting the capsule and Rnase P primers targeting the Human Rnase P for internal control of the experiment. We used CDC supplied primers PA-Forward: CGG ATC AAG TAT ATG GGA ATA TAG CAA, PA-Reverse: CCG GTT TAG TCG TTT CTA ATG GAT, PA-Probe: FAM-CTC GAA CTG GAG TGA AGT GTT ACC GCA AAT-BHQ1 and for Capsule; Cap-Forward: ACG TAT GGT GTT TCA AGA TTC ATG, Cap-Reverse: ATT TTC GTC TCA TTC TAC CTC ACC and Cap-Probe: FAM-CCA CGG AAT TCA AAA ATC TCA AAT GGC AT-BHQ1. The PCR mixture (25μL total reaction volume) contained 12.5μl of Promega mastermix (Promega Corporation, WI USA), 0.1μM of each probe and 0.3μM of each primer and 5μl of DNA template. Each primer set was run in a separate PCR tube. Template DNA was initially denatured by heating at 95°C for 10 min, followed by 40cycles of denaturation at 95°C for 15 sec, annealing and primer extension at 60°C for 1min using the Stratagene MX3000P Real-time PCR system. We interpreted a positive result as a sample that had all targets of Rnase-P and PA (pXO1) and Cap (pXO2 gene targets detected and a negative result was a sample were only the Rnase- P target was detected.

## Results

### Descriptive epidemiology

We identified 61 case-patients: 58 suspected and three confirmed. Of the 61 case-patients, 48 (79%) were hospitalized. The mean age of the case-patients was 30 years (*SD* = 20.23). The attack rate (AR) was the highest in the >65year age-group (0.38/1000). Males and females were similarly affected (Table 1).

**Table 1:** Attack rate by sex and age during a gastrointestinal anthrax outbreak: Isingiro, Uganda, August 2017.

Characteristics	Population	Cases	Attack rate / 1000
**Sex**			
Male	237,549	29	0.12
Female	254,568	32	0.13
**Age (years)**			
0–5	114,093	7	0.06
6–12	104,023	6	0.06
13–16	48,012	5	0.10
17–35	145,438	20	0.14
36–64	67,365	18	0.27
>65	13,185	5	0.38

**Fig 1:**
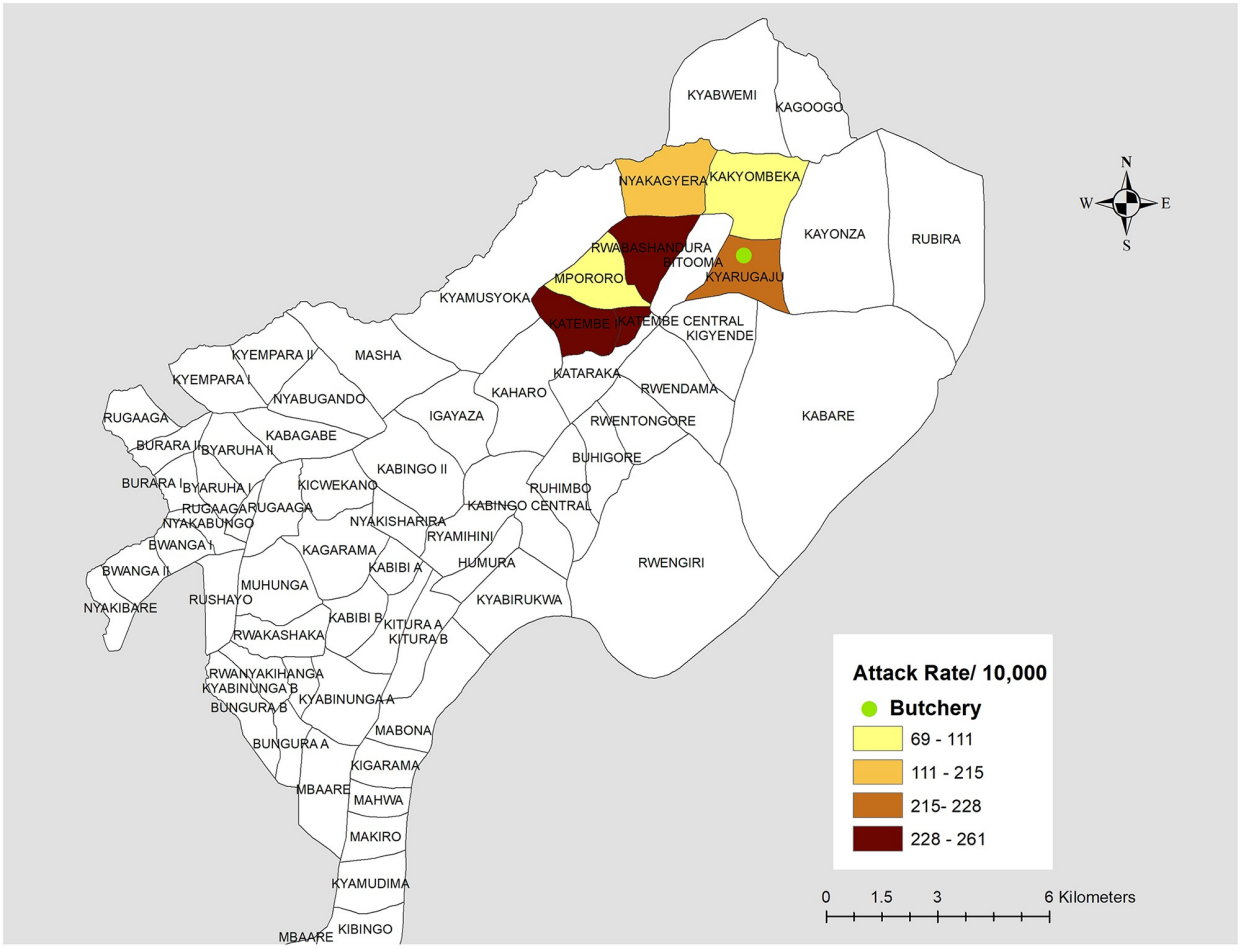
Attack rates by villages in Kabingo subcounty, Isingiro District, Uganda, August 2017 (map drawn using QGIS browser 2.8.2).

Descriptive epidemiology indicated that many of the case-patients reportedly had eaten beef purchased from a single vendor (butchery X), and this became the focus of our investigation. Of the 65 villages in Kabingo sub-county, six were affected, all of which are within a six-kilometer radius of butchery X (Fig 1).

Of the 61 cases, 50 had complete data on symptoms and exposures. The case-patients presented with gastrointestinal symptoms and fever (Table 2).

**Table 2:** Distribution of symptoms of suspected cases of gastrointestinal anthrax during an outbreak: Isingiro, Uganda, August 2017.

Symptom	Number of cases (N = 50*)	Percentage
Abdominal pain	47	94
Acute onset of diarrhoea	44	88
Non-bloody diarrhoea	37	74
Bloody diarrhoea	7	14
Vomiting	32	64
Fever (self-reported)	25	50

* Data on clinical symptoms were available for 50 of the 61 case-patients.

**Fig 2:**
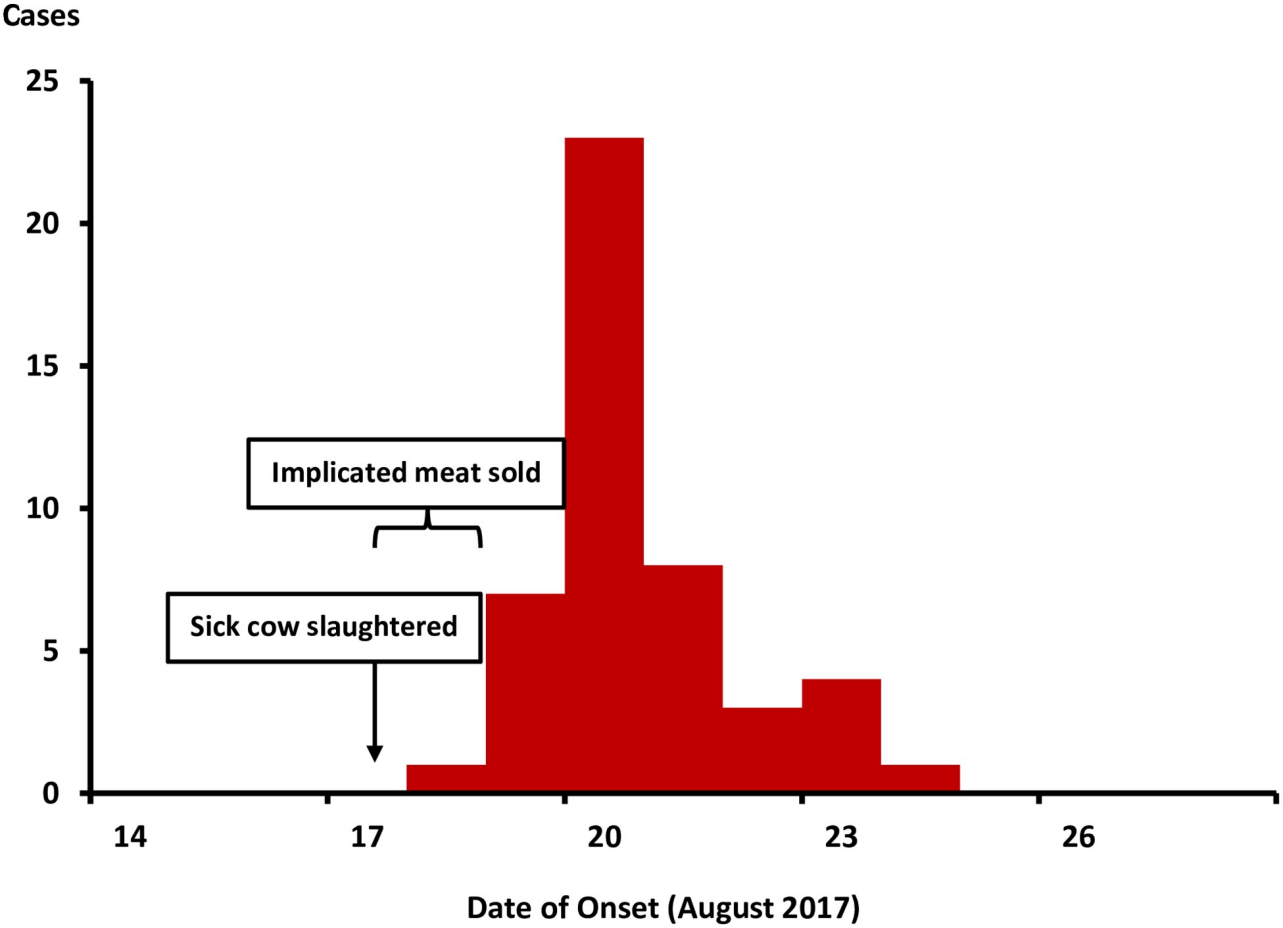
Distribution of symptom onset date of 61 gastrointestinal anthrax case-patients during an outbreak: Isingiro District, Uganda, August 2017.

The first case-patient became ill on 18 August (Fig 2). Case counts rapidly increased and peaked on 20 August, rapidly declined thereafter, and ended on 24 August. This epidemic curve was consistent with a point-source exposure.

Assuming the illness was gastrointestinal anthrax with a median incubation period of 3 days and a range from 1 to 6 days [3,12], we calculated the period of likely exposure to be from 17–18 August. Although cutaneous anthrax is known to commonly occur along with the gastrointestinal anthrax, we did not identify any case-patients with signs of cutaneous anthrax. The persons who reportedly skinned the animal–the butcher and his colleagues–had reportedly fled, and were unable to be located.

The attending clinician at MRRH had suspected a severe bacterial infection based on the patients’ symptoms (the bloody diarrhoea in some of the patients and severe dehydration), and had immediately initiated administration of intravenous antibiotic treatment for all patients including: metronidazole, amoxicillin and ciprofloxacin, and intravenous replacement with normal saline and Ringer’s lactate. No case-patient died.

### Case-control study results

The 50 case-patients with complete data on symptoms and exposure were included in the case-control study. The proportion of case-patients that ate meat exclusively from butchery X was 82% compared to 6.0% who did not eat beef at all (MOR = 46, 95% C.I: 4.7–446). On the other hand, 4.0% ate beef exclusively from other sources compared to 6.0% who never ate beef at all (MOR = 0.36, 95% C.I: 0.030–4.4) (Table 3).

**Table 3:** Association between source of meat and case status during a suspected gastrointestinal anthrax outbreak: Isingiro, Uganda, August 2017.

Beef eaten during 11–18 Aug	% Cases (n = 50)	% Controls (n = 100)	MOR (95% CI)*
Ate any beef			
Yes	94	70	8.8 (2.0–38)
No	6	30	Ref
Source of beef			
Butchery X only	82	12	46 (4.7–446)
Butchery X & elsewhere	8	3	19 (1.05–328)
Other sources only	4	55	0.36 (0.030–4.4)
Did not eat beef	6	30	Ref

### Trace-back investigation findings

Butchery X is located at Kyarugaju Village, and was in operation for >5 years before this outbreak. At the time of our investigation, the owner had closed butchery X, and declined repeated requests for interview. The butchery sold beef to case-patients on 17 and 18 August 2017 (Fig 1). No remnants of butchery X beef from that time were available for testing at the time of our investigation. Interviews with neighbours revealed that two kinds of meat were sold at butchery X: “cheap” meat and the “normal-priced” meat. Subsequent interviews with case-patients indicated that the “cheap meat” was the only beef sold at butchery X during the exposure period. Butchery X at that time sold the “cheap” meat at half the market price of meats in the area. According to interviews with the neighbouring butcheries and shops, the implicated “cheap” meat was reportedly obtained from a cow living on a farm in Rwefunjo Village, Masha sub-county, Isingiro District, approximately 7.5km north of the outbreak-affected area.

When we visited the farm, the farm owner reported that the cow in question had been ill prior to slaughter. Symptoms included general weakness, wasting, and lack of appetite. The cow had a spontaneous abortion four weeks before slaughter. The farm owner reported that a veterinarian treated the cow with injectable calcium and antibiotics. We were unable to determine which antibiotics were used. When symptoms did not improve, the cow was sold to the owner of butchery X. He slaughtered the cow at a location near the farm on 17 August 2017. Although the beef was not officially inspected, the owner of the cow and other community members who participated in the slaughter reported that it appeared normal, except for the liver, which appeared darker than normal. The beef was subsequently transported to butchery X for sale on 17 and 18 August 2017. These dates were consistent with the estimated likely period of exposure, based on calculations from the epidemic curve.

The farm owner reported that the other livestock on the farm were all in good health. However, on inspection, the investigation team found one cow with bloody diarrhea; a stool sample was collected from this cow. According to the District Veterinary Officer, livestock in the area had not been vaccinated against anthrax at least in the past five years. He reported no unexplained mass livestock die-outs in the district in recent history.

### Laboratory investigation results

Microscopy of the one vomitus sample showed square-edged, gram-positive, chained strepto-bacilli with empty spaces inside, morphologically consistent with *B*. *anthracis*. Microscopy of the samples at MRRH never showed any other bacterial pathogens. However, culture of one vomitus sample yielded growth of greyish white colonies of non-haemolytic bacteria whose gram stain was consistent with *B*. *anthracis*. At UVRI, two of the three human vomitus samples and one of the three stool samples were positive for *B*. *anthracis* by culture. The positive cultures of two samples of vomitus and one stool sample were further tested at UVRI, and found to be positive for *B*. *anthracis* by PCR. None of the eight blood samples demonstrated presence of *B*. *anthracis*. The stool sample from the cow with bloody diarrhoea was negative for *B*. *anthracis* by culture (Table 4).

**Table 4:** Laboratory results of specimen testing during gastrointestinal anthrax outbreak: Isingiro, Uganda, August 2017.

Source	*B*. *anthracis* Assay Result by Specimen Type		
	Blood	Vomitus	Stool
Case-patient 1	C-	M+, C+, PCR+	C-
Case-patient 2	C-	C-	C+, PCR+
Case-patient 3	C-	C+, PCR+	C-
Case-patient 4	C-		
Case-patient 5	C-		
Case-patient 6	C-		
Case-patient 7	C-		
Case-patient 8	C-		
Sick Cow (herd mate of implicated source; not the implicated cow)	C-		C-

The confirmed case-patients all belonged to the group that ate beef exclusively from butchery X and they were all between 30 and 35 years of age. However, each of the confirmed cases belonged to a different household.

## Discussion

Our investigation documented a gastrointestinal anthrax outbreak associated with eating beef in a district that is a major cattle producer in southwestern Uganda. The implicated beef came from a cow that was ill before slaughter, and the laboratory investigation identified anthrax in three case-patient specimens. The cow that was the source of the implicated meat did not have a typical presentation of anthrax infection, and a specimen sample from an ill cow on the same farm as this cow was negative for *B*. *anthracis*.

Anthrax is known to be endemic in eastern Africa, particularly in areas where humans, livestock, and wildlife interact; however, it is often underdiagnosed or underreported [7]. In Uganda, the only previously-documented human gastrointestinal anthrax outbreak was reported in 1976. That outbreak affected 143 persons, causing 9 deaths among children [17]. Similar to the current outbreak, the 1976 outbreak occurred after eating the meat of a cow that died of an unknown cause, and patients similarly presented with nonspecific initial symptoms and none of the patients presented with signs of cutaneous anthrax [17]. Atypical anthrax presentation has been documented in other previously published studies as well [11]. In a community gastrointestinal anthrax outbreak in Thailand, 91% of the case-patients presented with only acute diarrhoea [10]. The lack of pathogen-specific manifestations often leads to misdiagnosis and delayed treatment, resulting in high case-fatality rates [11]. During the current outbreak, the few patients with bloody diarrhoea alerted the clinicians of the possibility of a severe bacterial gastroenteritis and immediate aggressive treatment with intravenous antibiotics, which might have helped to explain the excellent patient outcomes. However, it is also possible that more than one pathogen has been involved in this outbreak. While the culture carried out on patient specimens would likely have identified other bacterial pathogens, if present, viral pathogens would not have been identified in this manner.

This outbreak occurred in August, which usually marks the end of the dry season and the start of the rainy season in this region [15]. This is consistent with literature that states that in some African eco-systems, anthrax outbreaks in livestock and subsequently in humans have been reported at the end of the dry season [7, 18] Such conditions force livestock to graze closer to the ground to reach the short and sparse grasses. In addition, the water bodies may collect and accumulate spores and so as livestock move closer to wildlife conservation areas in search of water, this may increase their risk of exposure to anthrax [7, 18]. In herbivores, per-acute anthrax is characterized by sudden death with bleeding from natural orifices and subcutaneous haemorrhages, fever, difficult breathing, agitation and convulsions, and septicemia on laboratory testing [7]. Often, cattle keepers recognize anthrax based on these symptoms [19]. However, cattle infected with anthrax may present atypically. For example, in 2011, during a fatal cattle gastrointestinal anthrax outbreak in Sheema District, Uganda, the cows did not present with typical features of anthrax [20]. During the current outbreak, there was no evidence of typical anthrax infection among other livestock on the farm where the suspected cow was raised.

Major limitations in this investigation included the inability to obtain remains of the implicated meat for testing, and that we were unable to interview the proprietor of butchery X to gather further information. As a result, we were unable to prove conclusively that the source of *B*. *anthracis* in this outbreak was the meat from the implicated cow, even though that was the most likely explanation for this outbreak. In addition, we did not use more robust tests such as the M’Fadyean or the Azur B stain in the initial microscopy testing as this would have added on the credibility of the diagnosis of *B*. *anthracis*. Also doing PCR sequencing on the stool sample of the cow that had bloody diarrhoea on the farm would have been more confirmatory that this sample was indeed negative for *B*. *anthracis*.

### Recommendations

After the investigation, we recommended enforcing inspection of all livestock prior to slaughter, health education about anthrax and its cause, dissuading residents from buying “cheap” meat, safe disposal of the carcasses of livestock or game animals, and vaccination of all cattle. Isingiro District implemented the first three of the recommendations, and is currently exploring ways to safely dispose of livestock carcasses and to mass-vaccinate livestock against anthrax.

Generally, anthrax outbreaks have both public health and economic aspects entwined in a social context. These need to be collectively addressed in endemic and epidemic prone areas such as Uganda [8,19]. For example, food security worsens as the dry season progresses, forcing people to salvage any meat available to them, including that of animals that have died as a result of unknown causes. To control future outbreaks, a One-Health approach, involving ministries responsible for human health, livestock, and wildlife services, should be considered [1]. In Uganda as elsewhere, the ministries of health, agriculture and wildlife should collectively conduct regular analysis of surveillance data from livestock, wildlife, and humans. Prevention and control measures should be aimed at livestock (for example, livestock vaccination and safe disposal of animal carcasses), human communities (i.e., stopping the eating of meat from animals that have died of unknown causes), and the environment (i.e., decontamination of sites where animals died) [13].
